# Ratiometric Activatable Cell-Penetrating Peptides Provide Rapid In Vivo Readout of Thrombin Activation**

**DOI:** 10.1002/anie.201205721

**Published:** 2012-10-18

**Authors:** Michael Whitney, Elamprakash N Savariar, Beth Friedman, Rachel A Levin, Jessica L Crisp, Heather L Glasgow, Roy Lefkowitz, Stephen R Adams, Paul Steinbach, Nadia Nashi, Quyen T Nguyen, Roger Y Tsien

**Affiliations:** Department of Pharmacology, UCSD School of MedicineUniversity of California San Diego, La Jolla, CA (USA); Department of Chemistry and BiochemistryUniversity of California San Diego, La Jolla CA (USA); Howard Hughes Medical InstituteLa Jolla, CA (USA); Division of Otolaryngology-Head and Neck Surgery, UCSD School of MedicineUniversity of California San Diego, La Jolla, CA (USA)

**Keywords:** activatable cell-penetrating peptides, FRET, peptides, ratiometric imaging, thrombin

Extracellular proteases including thrombin are involved in numerous biological processes and play major roles in a variety of human diseases. The spatial and temporal patterns of activation of proteases in vivo control their biological role in diseases and amenability to therapeutic targeting. Previously we developed activatable cell-penetrating peptides (ACPPs) to monitor matrix metalloproteinase (MMP) and elastase activity in tumors. Later ACPPs detect thrombin activation in atherosclerosis and brain injury. We have now modified the thrombin ACPP in two independent ways, 1) to provide a FRET-dependent emission ratiometric readout and 2) to accelerate the kinetics of cleavage by thrombin. Emission ratioing improves kinetic detection of enzyme activity, because it reflects the ratio of cleaved versus uncleaved probe but cancels out total probe concentration, illumination intensity, detection sensitivity, and tissue thickness. Because pharmacokinetic washout of the uncleaved probe is not necessary, yet the cleavage converts a diffusible substrate into an immobilized product, thrombin activity can be imaged in real time with good spatial resolution. Meanwhile, placement of norleucine-threonine (Nle-Thr) at the P4-P3 substrate positions accelerates the kinetics of thrombin cleavage by 1–2 orders of magnitude, while preserving selectivity against related proteases. The new ratiometric ACPPs detect localized thrombin activation in rapidly forming blood clots minutes after probe injection, and the signal is inhibited by thrombin specific inhibitors.

Thrombin is a serine protease and a key regulator of blood coagulation. It is responsible for the proteolytic cleavage and activation of multiple coagulation factors including Factor V, VIII, XI, as well as fibrinogen and protein C.^1^–^2^ Thrombin also cleaves and activates protease-activated receptors (PARs) which are highly expressed on platelets, endothelial cells, myocytes, and neurons.^3^–^5^ Thrombin is a major therapeutic target for thrombosis and stroke intervention/prevention through indirect inhibitors such as heparin or warfarin, and direct inhibitors hirudin (divalent), and argatroban (monovalent).^6^, ^7^. In addition to its role in thrombosis and stroke,^8^–^11^ thrombin is reported as a relevant player in cardiovascular disease,^12^, ^13^ renal injury,^14^ and cancer.^15^ Activatable cell-penetrating peptides (ACPPs) target various cargos, including fluorescent imaging agents, to sites of protease activity in vivo.^16^–^19^ ACPPs consist of a polycationic cell-penetrating peptide attached to a cargo and a polyanionic inhibitory domain with a protease-cleavable linker. Probe activation and cargo uptake depend on localized proteolysis of the linker sequence that connects the polyanionic and polycationic domains, which converts the probe to an adherent form. This method provides detection of spatially localized enzymatic activity in living tissues through the accumulation of cleaved probe. ACPPs have been previously reported that target MMPs^16^, ^17^ and elastases 20 in tumors. A thrombin-activated ACPP with cleavage sequence DPRSFL, from the PAR1 receptor was recently reported for monitoring thrombin activation in atherosclerotic plaques.^21^ This ACPP is efficiently cleaved by thrombin and accumulates in atherosclerotic plaques with increasing signal depending on plaque load. An optimized and more selective thrombin-cleavable ACPP with a substrate sequence of PPRSFL has also been used to measure thrombin activation after brain injury.^22^ Each of these ACPPs included a single fluorophore (Cy5) and therefore quantitative measurement required time to allow uncleaved peptide to wash out of the target tissue before the contrast could be seen. Probes based on fluorescence dequenching have previously been used to detect thrombin activity during clot formation, but many factors other than enzyme activity also affect fluorescence intensity, and diffusion of the agent after cleavage limits signal intensity at the site of protease activation.^23^ In this report, we describe a new ratiometric ACPP that combines the triggered retention inherent to ACPPs with the advantages of spectral imaging to detect spatial and temporal changes in thrombin activity in vivo within minutes of tail amputation. We also disclose a new substrate sequence that is cleaved by thrombin 1–2 orders of magnitude faster than its predecessors, derived from protease-activated receptor-1 (PAR-1), one of the most important natural thrombin substrates.

Ratiometric ACPPs (RACPP, structures **5**, **10**, **15, 20**, and **25** in the Supporting Information) differ from non-ratiometric ACPPs by the attachment of a fluorescent acceptor such as Cy7 to the polyanionic domain so that in the intact, uncleaved probe, Cy5 on the polycationic domain undergoes efficient fluorescence resonance energy transfer (FRET) to the acceptor fluorophore (Figure 1 a). Upon linker cleavage by thrombin, the resulting separation of the polyanionic and polycationic sequences disrupts FRET, instantly restoring the Cy5 fluorescence (peak at approximately 670 nm) and eliminating the Cy7 re-emission (peak at approximately 780 nm). The Cy5 attached to the CPP portion of the probe is retained at the site of cleavage so that its dequenched emission remains localized. In vitro, the addition of purified thrombin to an RACPP with substrate sequence PPRSFL (RACPP_PPRSFL_), diluted in plasma, resulted in a 34-fold change in the Cy5/Cy7 emission ratio. This ratio change is the result of an 8.8-fold increase in Cy5 emission (Figure 1 b, blue line) and a 3.8-fold decrease in Cy7 re-emission (Figure 1 b, red line). The initial thrombin-cleavable ACPP used the substrate sequence DPR↑SFL, amino acid residues 39–44 of the thrombin receptor PAR-1, and in which ↑ marks the site of cleavage. The PPRSFL cleavage sequence was identified by substitution mutagenesis as a more selective thrombin substrate. The substitution of proline at the P3 position to increase specificity for thrombin over plasmin is consistent with results from previous positional scanning reports.^24^ Kinetic analysis was used to determine the susceptibility of DPRSFL (**5**) and PPRSFL (**10**) RACPPs to thrombin, plasmin, and factor Xa, the protease that activates prothrombin. In vitro measurements yielded *k*_cat_/*K*_m_ = 1.2×10^4^ m^−1^ s^−1^ for thrombin with RACPP_DPRSFL_, compared to the previously reported *k*_cat_/*K*_m_ of 2.1×10^4^ m^−1^ s^−1^ for the non-ratiometric DPRSFL ACPP.^21^ However, the *k*_cat_/*K*_m_ values for plasmin (1.0×10^4^ m^−1^ s^−1^) and factor Xa (6.2×10^3^ m^−1^ s^−1^) were less than 2 fold different than thrombin. In contrast, RACPP_PPRSFL_ (**10**) showed a slightly lower *k*_cat_/*K*_m_ (7.3×10^3^ m^−1^ s^−1^) for thrombin but much greater selectivity over plasmin (14.3-fold lower *k*_cat_/*K*_m_) and factor Xa, which showed no detectable activity towards RACPP_PPRSFL_. To confirm that the spectroscopic readout was due to peptide cleavage, the RACPPs (**5**, **10**, and **20**) were incubated with enzyme and separated using SDS-polyacrylamide gel electrophoresis (Figure 2). These gels were analyzed using multispectral imaging (*λ*_ex_ = 620, *λ*_em_ = 640–840 nm) and displayed as the ratio of Cy5 (approximately 680 nm) to Cy7 (approximately 780 nm) emissions in pseudocolors from blue (ratio minimum) to red (ratio maximum) using custom-designed software. This direct ratiometric imaging visually distinguishes uncleaved probe, in which FRET was intact (Figure 2, blue), from cleaved probe, in which FRET was disrupted (Figure 2, red). Images confirmed that RACPP_PPRSFL_ (**10**) and RACPP_DPRSFL_ (**5**) are cleaved by thrombin in a time-dependent manner and that RACPP_PPRSFL_ (**10**) is selective for thrombin. An MMP cleavable RACPP_PLGC(Me)AG_ (**20**) was also shown, as a control that was not cleaved by any of the pro-coagulation enzymes. Because SDS-PAGE did not separate intact RACPP from Cy7-anionic fragments, we developed buffer conditions using pentaethylenehexamine (PEHA)-acetate and agarose gels that showed distinct bands for all the three expected components (Supporting Information, Figure S1).

**Figure 1:**
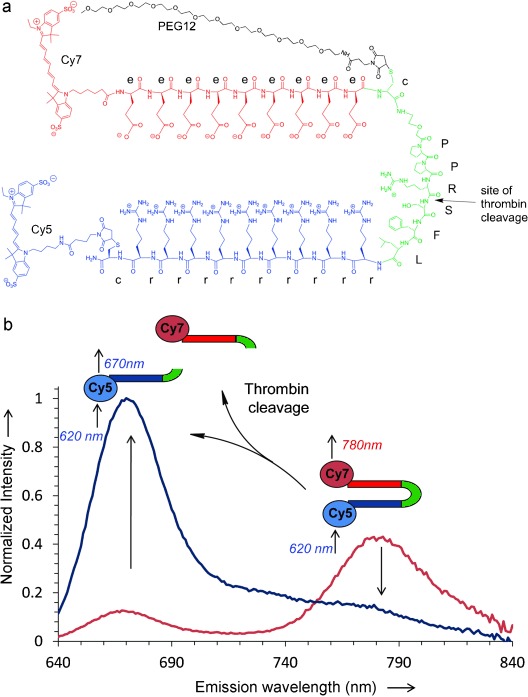
a) Chemical formula of RACPP_PPRSFL_. A polyanionic domain (red) is connected with a thrombin-cleavable linker (PPRSFL or NleTPRSFL) (green), to a polycationic domain (blue), conjugated to Cy5. c = d-cysteine; e = d-glutamate; r = d-arginine. b) Emission spectrum of RACPP_PPRSFL_, measured in mouse plasma in a cuvet spectrofluorometer, before (red) and after (blue) treatment with thrombin. Before cleavage, Cy5 is quenched by Cy7, which re-emits at 780 nm. After cleavage, Cy7 no longer quenches Cy5, so the 670 nm peak from Cy5 increases and Cy7 re-emission disappears. The residual shoulder from 710 nm to 840 nm is largely from Cy5 emission.

**Figure 2:**
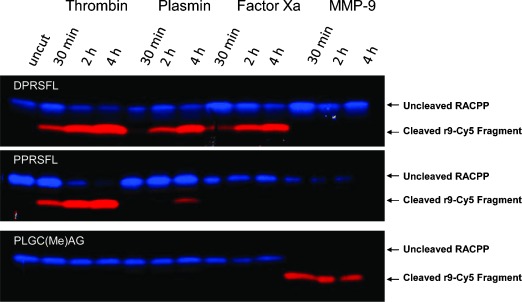
Peptide cleavage of RACPPs by purified thrombin, plasmin, factor Xa, and MMP-9. Three RACPPs (top: DPRSFL, middle: PPRSFL, and bottom: PLGC(Me)AG) were separately exposed to purified enzymes for the times indicated. Peptide cleavage products were separated by electrophoresis using tricine-SDS polyacrylamide gels and imaged using the Maestro with 620 nm excitation, and emission collected for Cy5 (660 to 720 nm) or Cy7 (760 to 830 nm). Ratiometric images were produced by dividing the Cy5 emission with Cy7 emission and pseudocolored from blue (ratio minimum) to red (ratio maximum).

To test the specificity of the RACPP_PPRSFL_ (**10**) in vivo, clot formation was monitored using a tail-clip model. Ten nanomoles of probe were injected intravenously into an adult mouse and the fluorescence signal was monitored over time in clotting blood exuded from a tail wound. After probe administration, ratiometric Cy5/Cy7 imaging was performed immediately, 10, 20, and 40 minutes post injury (Figure 3 a, b). The fluorescence ratio showed a rapid (within ten minutes) localized increase at the wound site (arrows), which continued to rise throughout the duration of clotting (max ratio change approximately 4.0; blue line). The spatial distribution of fluorescence was not diffuse, but rather showed a gradient, suggestive of a higher thrombin concentration in the blood closest to the wound. RACPP_PPRSFL_ (**10**) was also tested in blood clots from mice that had been pre-injected with the direct and selective thrombin inhibitor hirudin. Addition of hirudin inhibited the ratio increase by >90 % (Figure 3 b, red line), which supports the conclusion that the signal in the developing clots is largely thrombin dependent.

**Figure 3:**
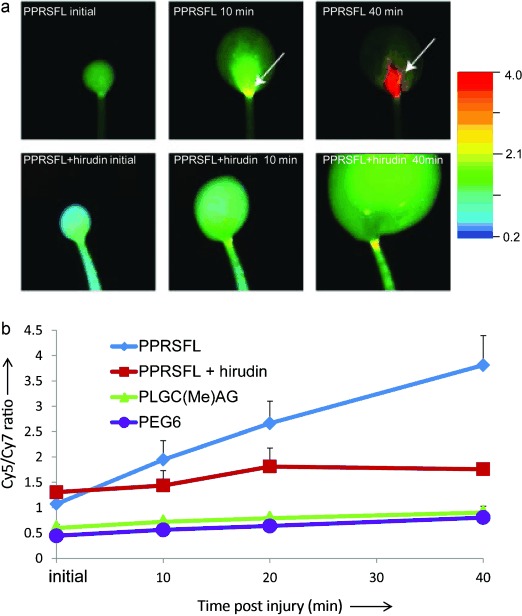
Detection of thrombin activity in developing blood clots. a) Ratiometric fluorescent images showing changes in Cy5/Cy7 emission ratios from tail clots of mice that had been injected with either RACPP_PPRSFL_ (top) or RACPP_PPRSFL_ with prior injection of hirudin (bottom). Images were taken 5, 10, and 40 min post injury (left to right). A Cy5/Cy7 ratio increase was detectable at 10 min as shown by the arrow (top, middle) and reached fourfold by 40 min (top, right) for RACPP_PPRSFL_. Pre-treatment with hirudin attenuates the Cy5/Cy7 ratio changes (bottom). b) Graph showing quantitative analysis of images shown in Figure 3 a with additional animals that were injected with either RACPP_PPRSFL_ (*n* = 5, blue), RACPP_PPRSFL_ with hirudin (*n* = 3, red), RACPP_peg6_ (*n* = 3, purple) or RACPP_PLGC(Me)AG_ (*n* = 4, green). Fluorescent intensities were acquired from identical rectangular areas from the region of interest (ROI), see arrows, using ImageJ. Cy5 fluorescent intensities were divided by Cy7 intensities and the ratios were plotted as the mean±SD for each treatment group.

Not surprisingly, the blood pool in the hirudin treated animals grew faster and the mice bled longer, as normal coagulation was inhibited (Figure S2). Likewise, control RACPPs that were cleavable by MMPs (RACPP_PLGC(Me)AG_, **20**) or uncleavable (**15**), with a poly(ethyleneglycol) linker of matching length (-HN(CH_2_CH_2_O)_6_CH_2_CH_2_CO-, “peg6”), maintained low and stable Cy5/Cy7 emission ratios at the wound site over 40 minutes of clotting (Figure 3 b, green and purple lines). The addition of purified thrombin or MMP-9 to the developing blood pools from mice that had been injected with either RACPP_PPRSFL_ (**10**) with hirudin or RACPP_PLGC(Me)AG_ (**20**) produced spectacular local ratiometric contrast (Figure S2). This verified the cleavability of the RACPPs for their respective enzyme in each of the negative controls.

Although RACPP_PPRSFL_ is currently our most characterized thrombin-selective ACPP, we have recently identified a new RACPP in which PPRSFL is replaced with norleucine-TPRSFL thereby accelerating thrombin cleavage approximately 90-fold. The new substrate combines the P4 to P1 residues (norleucine-TPR↑), identified by positional scanning,^24^ with the P1′ to P3′ amino acids (SFL) from PAR-1 and our previous ACPPs. RACPP_NleTPRSFL_ (**25**) had a *k*_cat_/*K*_m_ = 6.7×10^5^ m^−1^ ^−1^, compared to 7.3×10^3^ m^−1^ s^−1^ for RACPP_PPRSFL_ (**10**), while maintaining 52.5-fold selectivity over plasmin (*k*_cat_/*K*_m_ = 1.3×10^4^ m^−1^ s^−1^) and 27.7-fold selectivity over factor Xa (*k*_cat_/*K*_m_ = 1.3×10^4^ m^−1^ s^−1^). To verify the accelerated cleavage, 1 μm RACPP_NleTPRSFL_ (**25**) or RACPP_PPRSFL_ (**10**) were incubated with thrombin (concentrations of 25 nm to 0.4 nm) for 30 minutes followed by analysis using gel electrophoresis and imaging (Figure 4 a). The percent cleavage of RACPP_NleTPRSFL_ by 0.4 nm thrombin was similar to the percent cleavage of RACPP_PPRSFL_ by 25 nm thrombin, consistent with the ratio of *k*_cat_/*K*_m_ for the two substrates. In addition, in the tail-clot model described above, RACPP_NleTPRSFL_ (**25**) gave a Cy5/Cy7 ratio increase from 0.75(±0.11) at one minute to 3.1(±0.45) at five minutes post injury (*n* = 3, *p* = 0.01; Figure 4 b) compared to no significant change in Cy5/Cy7 ratio for RACPP_PPRSFL_ over the same time interval (Figure 3, Figure S3). At 15 minutes post injury, the Cy5/Cy7 ratio increased to 7.2 (a tenfold change) for RACPP_NleTPRSFL_ compared to less than 2.5-fold for RACPP_PPRSFL_. Co-administration of lepirudin, a clinically approved recombinant analogue of hirudin, inhibited the cleavage and ratio change of RACPP_NleTPRSFL_ as expected (Figure 4 b, bottom row), confirming that the response was thrombin dependent.

**Figure 4:**
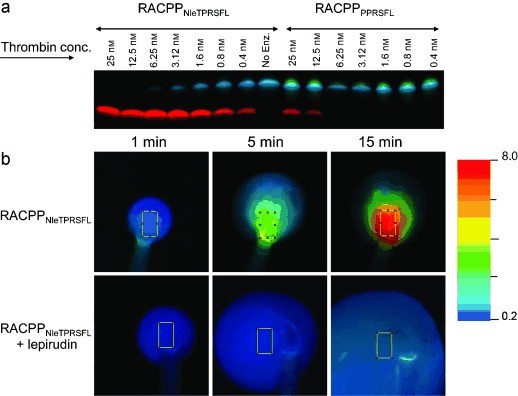
a) Peptide cleavage of RACPP_NleTPRSFL_ and RACPP_PPRSFL_ by purified thrombin at concentrations from 25 nm to 0.4 nm. Peptide cleavage products were separated by electrophoresis and imaged at *λ*_ex_ = 620 nm and the emission collected for Cy5 (660 to 720 nm) and Cy7 (760 to 830 nm). Ratiometric images were produced by dividing the Cy5 and Cy7 emission intensities and pseudocoloring. b) Ratiometric fluorescent images showing changes in Cy5/Cy7 emission ratios from tail clots of mice that had been injected with RACPP_NleTPRSFL_ (top) or RACPP_NleTPRSFL_ with lepirudin (bottom). For quantification, average Cy5 and Cy7 fluorescent intensities were acquired from identical ROI (highlighted in figure) and divided to determine the Cy5/Cy7 ratios.

We have used the earliest generation thrombin-activated RACPP_DPRSFL_ (**5**) to image thrombin activity in atherosclerotic plaques in carotid and aortic arteries in vivo. Thrombin activity was first visualized in atherosclerotic plaques located in the carotid artery, which was surgically exposed in live mice under conditions of normal blood flow (*n* = 2). Mice were imaged under white light (Figure 5 a) followed by direct ratiometric imaging 2.5 hours after probe injection. Thrombin activity was detectable in plaques that could be seen with white light (Figure 5 a) with the most intense signal correlating to plaques localized on the carotid bifurcation (Figure 5 b). Animals were then euthanized prior to dissection to expose the aortic arch and the lower carotid. Again, white light (Figure 5 c) and ratiometric (Figure 5 d) images are shown. The aortic arch, brachiocephalic trunk, and carotid arteries all showed significant plaque load by white light. Interestingly, high thrombin activity correlated to only sub-regions of the plaques and not necessarily to the regions with the thickest plaques. Detailed analysis to correlate thrombin activation with disease pathology^21^ will be required to understand the disease significance of localized thrombin activation within plaques. Future studies on these atherosclerosis models will be performed with RACPP_NleTPRSFL_ (**25**).

**Figure 5:**
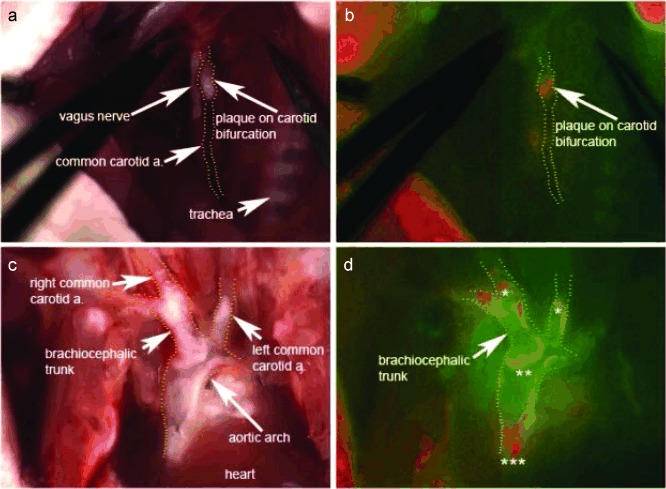
a) In vivo imaging of the carotid showing an atherosclerotic plaque at the bifurcation with white light reflectance. b) Fluorescence imaging of the same carotid artery (stippled lines) showing variable Cy5/Cy7 ratios within the visible plaque, indicating heterogeneity within the plaque. Note that the vagus nerve immediately adjacent to the carotid artery has a similar whitish opacity as the plaque within the carotid bifurcation on white light reflectance but can be easily differentiated with fluorescence imaging by its lack of probe uptake. c) White light image showing extensive atherosclerotic plaques at the level aortic arch, brachiocephalic trunk, and bilateral carotid arteries. d) Fluorescence ratio imaging of the same vessels (highlighted) showing variable Cy5/Cy7 ratios indicating heterogeneity in thrombin activity in visible plaques. Cy5/Cy7 ratios were low (green) for plaques at the mid-level of the brachiocephalic trunk (arrow) compared to plaques with higher ratios (orange/red color = higher thrombin activity) at the root of the aorta (three asterisks), brachiocephalic trunk (two asterisks), or within the right and left carotid arteries (single asterisks).

Ratiometric ACPPs selectively cleaved by thrombin provide a sensitive probe for monitoring physiologically activated thrombin in real time. FRET from Cy5 to Cy7 within an ACPP provides a significant improvement over intensity-based ACPPs or fluorescence-dequenching probes by eliminating the need for washout to generate contrast at the site of cleavage and canceling many nonenzymatic factors that perturb intensity measurements at single wavelength bands. Although FRET substrates for proteases have long been popular, the combination of ACPPs with FRET is novel and brings many important advantages. Enzymatic cleavage not only produces a large spectroscopic shift but also converts a diffusible substrate into an adhesive product, which remains localized at the site of cleavage to confer spatial resolution. The hairpin structure holds the Cy5 and Cy7 at a distance conducive to FRET rather than hydrophobically driven stacking and mutual static quenching. Therefore, cleavage causes a large (approximately 40x) change in emission ratio regardless of the substrate sequence or enzyme being sensed. The long wavelengths of Cy5 and Cy7 are ideal for in vivo imaging, where excitation and emission wavelengths should be well above 600 nm to avoid the strong absorbances of endogenous hemes. These properties are all optimized for the demanding application of in vivo imaging with high spatial and temporal resolution using a minimum probe concentration. For strictly in vitro assays, much smaller and simpler molecules may suffice.^25^

A thrombin-dependent ratio increase was detected with RACPP_PPRSFL_ (**10**) less than ten minutes after tail wounding. RACPP_NleTPRSFL_ (**25**) showed an even larger response at five minutes. Accurate assessment of the performance improvement will require testing in more sophisticated and clinically relevant models. Further optimization should also be possible, because several recent reports describe potential new thrombin-selective substrates that could be incorporated into RACPPs to attempt to increase both sensitivity and specificity.^5^, ^25^

Thrombin activation is dynamically regulated in clotting blood and continues to be active even when bound to fibrin after the clot has formed. Previous work with a near-infrared fluorescent (NIRF) dequenching probe demonstrated diffuse and rapid thrombin activation within 12 minutes of tail clipping, although thrombin specificity was not tested with pharmacological inhibitors.23a Signal from the dequenching probe was dispersed throughout the clot whereas our results show the highest ratio closest to the wound site, possibly because of localized thrombin and probe retention at the site of cleavage. Thrombin-specific antibodies can be used to localize thrombin and prothrombin antigen, but immunohistochemistry is destructive and static, and immunoreactivity does not necessarily correlate with proteolytically active thrombin. We were also able to demonstrate that the FRET probe is consistently and significantly protected from protease cleavage when the direct thrombin inhibitors hirudin or lepirudin are co-administered. Further studies are needed to test this probe in clinically relevant clots such as deep vein thrombosis and stroke.

Preliminary studies show that the first generation RACPP_DPRSFL_ (**5**) can sensitively detect thrombin activity in sub-regions of atherosclerotic plaques in the aorta and carotid arteries. Other clinical methods such as magnetic resonance imaging (MRI) or ultrasound can easily measure plaque burden but accurate clinical staging of plaques typically requires post mortem pathological analysis. Our previous report showed increased fluorescence uptake of non-ratiometric thrombin-cleavable ACPP in plaques with histologic features associated with more advanced disease from human studies.^21^, ^26^–^27^. The non-ratiometric thrombin ACPPs also showed a correlation between thrombin activity and the severity and spatial extent of damage in the ischemic core of stroke.^22^ The ratiometric ACPPs should be even better for such studies on disease etiology, because they signal thrombin activity more quickly and more reliably. For example, some zones of high enzyme activity may be so poorly perfused as to be inaccessible to the probe. With a non-ratiometric probe, those zones would be indistinguishable from regions with perfusion but low enzyme activity. With a ratiometric probe, inaccessible zones would have no signal at either wavelength, whereas perfused regions with low enzyme activity would show strong FRET and thus be clearly distinguishable.

There are three potential extensions to clinical applications to be considered. Analogous ACPPs attached to dendrimers labeled with Gd chelates have given MRI contrast for primary tumors^18^ and metastatic lymph nodes,^31^ so we are analogously attempting to image thrombin activity in atherosclerotic plaques with MRI. Unfortunately, MRI does not have ready equivalents for FRET or ratiometric fluorescence imaging. Endoscopic catheters can now image within arteries,^28^ so optical discrimination of atherosclerotic plaques from inside the artery could be valuable, especially if a correlation between thrombin activity and plaque vulnerability could be validated. Imaging of plaques from outside the artery, as in Figure 5, could be valuable during surgery, either to graft a bypass or to remove a nearby tumor, when it is important to avoid disturbing the plaque.

## Experimental Section

Synthesis of RACPPs: RACPPs (**5**, **10**, **15**, **20**, and **25**) were prepared by Fmoc solid-phase peptide synthesis and followed the same synthetic procedures that were used for elastase or MMP cleavable RACPPs.^29^ Peptides were purified using preparative HPLC and characterized using analytical HPLC, combined with mass spectrometry. Details of the synthesis and characterizations are shown in the Supporting Information.

Animals and in vivo testing in clots: Mice were anesthetized with ketamine/xylazine and RACPPs (10 nmol) were administered by way of bilateral retro-orbital injections. This study reports data from mice that have been injected with either RACPP_PPRSFL_ (with or without hirudin), RACPP_peg6_ (**15**), or RACPP_PLGC(Me)AG_ (**20**). RACPP_PLGC(Me)AG_ is closely related to RACPP-2 described by Savariar et al.^29^ for imaging MMP-2/-9 activities in tumors and metastases, but RACPP_PLGC(Me)AG_ adds a solubilizing PEG12 chain attached by way of a d-Cys following the polyglutamate sequence. For thrombin inhibition studies, mice were injected subcutaneously with hirudin (2000 U/mouse; *n* = 3) 20 min prior to probe injection. Immediately after probe injection, the tail was amputated 2.5 mm from its tip and the mouse was placed in the Maestro imager. Lepirudin (Refludan, Bayer) was administered at 0.5 mg/mouse immediately prior to injection of RACPP_NleTPRSFL_ (**25**). Multispectral images were acquired by exciting Cy5 at 620±10 nm and collecting the emitted light through a tunable liquid crystal filter from 640 nm to 840 nm with 10 nm step size. Cy5 and Cy7 emission images were generated by integrating from 660 to 720 nm (Cy5) and 760 to 830 nm (Cy7). Ratiometric images were produced by dividing the Cy5 emission by the Cy7 emission and creating pseudocolor from blue (ratio minimum) to red (ratio maximum) using custom-designed software. The absolute brightness in the ratiometric images was encoded from the corresponding Cy5 image. For image display, all images were identically scaled for the ratio linearly increasing from 0.2 (blue) to 4.0 (red), except for Figure 4, in which the scale was 0.2 (blue) to 8.0 (red). Significance was assessed using an unpaired two-tailed Student’s t-test.

Animals and in vivo testing in atherosclerosis: ApoE-/- mice (Jackson Laboratory) were in a C57/BL6 background and had been backcrossed 10 times. Mice were fed a 0.5 % cholesterol diet (Harlan Laboratories) for 3–6 months.^30^ Intraoperative imaging of atherosclerotic plaques was performed 2.5 h after intravenous injection of 10 nmol of RACPP_DPRSFL_ (**5**). Prior to imaging, animals were anesthetized with ketamine/xylazine (100 mg kg^−1^, 10 mg kg^−1^) and the carotid arteries were exposed. Other structures, including the carotid bifurcation and the aortic arch, were exposed post mortem. All structures were imaged using a customized fluorescence dissecting microscope (Olympus MVX) with two cameras sampling simultaneously. Excitation was at 615–645 nm, while Cy5 emission was collected from 665–705 nm and Cy7 emission from 754–816 nm. The ratio of Cy5 to Cy7 emissions was calculated in real time and displayed as described above for the Maestro-derived images.

All animal procedures were approved by UCSD’s Institutional Animal Care and Use Committee.

## Notes

Detailed facts of importance to specialist readers are published as ”Supporting Information”. Such documents are peer-reviewed, but not copy-edited or typeset. They are made available as submitted by the authors.
