# Supplementary material for: Ratiometric Activatable Cell-Penetrating Peptides Provide Rapid In Vivo Readout of Thrombin Activation
Source: Angew Chem Int Ed Engl. 2012 Oct 18;52(1):325–30. doi: 10.1002/anie.201205721 (PMC3694763; doi:10.1002/anie.201205721)
Supplement: Supplementary file 1 [file anie0052-0325-sd1.pdf]

Angewandte Chemie
Eine Zeitschrift der Gesellschaft Deutscher Chemiker

Supporting Information

© Wiley-VCH 2012

69451 Weinheim, Germany

Ratiometric Activatable Cell-Penetrating Peptides Provide Rapid In Vivo Readout of Thrombin Activation**

*Michael Whitney, Elamprakash N. Savariar, Beth Friedman, Rachel A. Levin, Jessica L. Crisp, Heather L. Glasgow, Roy Lefkowitz, Stephen R. Adams, Paul Steinbach, Nadia Nashi, Quyen T. Nguyen, and Roger Y. Tsien**

anie_201205721_sm_miscellaneous_information.pdf

General reagents and methods:

All the reagents and solvents were obtained from commercial sources and used without further purification. All the reactions involving Cy5 and Cy7 were carried out under dark or shielded from light. HPLC characterizations and purifications were performed on Agilent 1100 or 1200 with reverse-phase C_{18} column (Phenomenex) using acetonitrile and water solvent system with 0.05% TFA as additive. Low resolution electrospray ionization (ESI) mass spectrometry was performed using Agilent HPLC connected to an Agilent LCMS trap XCT. UV absorbance was recorded on Cary 3E (Varian) or UV-2700 (Shimadzu). Fluorescence was recorded in quartz cuvets using a spectrofluorometer (FluoroLog®, Horiba Scientific).

All the RACPPs were pegylated with mPeg12 to prevent aggregation and to improve solubility

Syntheses of peptides:

Solid phase peptide synthesis and HPLC purification

↓

NH_2-e9-c(S-S*t*Bu)-(Substrate)-r9-c-$CONH_2$ (**1, 6, 11, 16, 21**)

↓ Cy5-Mal, NMM, DMSO

NH_2-e9-c(S-S*t*Bu)-(Substrate)-r9-c(Cy5)-$CONH_2$ (**2, 7, 12, 17, 22**)

↓ Et_3P

NH_2-e9-c(SH)-(Substrate)-r9-c(Cy5)-$CONH_2$ (**3, 8, 13, 18, 23**)

↓ Mal-Peg12, NMM, DMSO

NH_2-e9-c(S-Peg12)-(Substrate)-r9-c(Cy5)-$CONH_2$ (**4, 9, 14, 19, 24**)

↓ Cy7-NHS, NMM, DMSO

Cy7-NH-e9-c(S-Peg12)-(Substrate)-r9-c(Cy5)-$CONH_2$ (RACPPs **5, 10, 15, 20, 25**)

1-5, Substrate = oDPRSFL
6-10, Substrate = oPPRSFL
11-15, Substrate = peg6
16-20, Substrate = oPLGC(Me)AG
21-25, Substrate = oNLeTPRSFL

Scheme 1: General method for the synthesis of RACPP

Synthesis of RACPP$_{DPRSFL}$: According to *Scheme 1*, NH_2-e9-c(SS-*t*Bu)-oDPRSFL-r9-c-$CONH_2$ (**1**) was prepared using conventional solid phase Fmoc peptide synthesis (Prelude, Peptide

Technologies, Inc), where lower case letters refer to D-amino acids, “o” denotes 5-amino-3-oxopentanoyl (a short hydrophilic spacer), C(Me) denotes S-methylcysteine and the final $CONH_2$ indicates C-terminal amide. The peptide was isolated from the solid support by treating it with mixtures of 92% trifluoroacetic acid (TFA), 2% thioanisole, 2% water and 4% triisopropylsilane (TIPS) for 5 h under N_2 atmosphere and filtered. This filtrate was concentrated and then precipitated by addition of ice cold 50% hexanes in methyl *tert*-butyl ether mixture. Centrifugation was performed to isolate the pellet that was dried under high vacuum. The peptide was dissolved in dimethyl sulfoxide (DMSO) and purified by high performance liquid chromatography (HPLC) using 5-55% acetonitrile in water and 0.05% TFA. The purified product (**1**) was dried by lyophilization (mass obtained 3695.7 Da, mass calculated 3696.1 Da).

To the purified compound **1** (20 mg) was added anhydrous DMSO (1 mL) Cy5 maleimide (Cy5-Maleimide, ~ 3 mg, synthesized from the NHS ester[1] by reaction with 2-aminoethylmaleimide and *N*-methylmorpholine (NMM) in DMSO, followed by precipitation with ethyl acetate), NMM (1μL) under N_2 atmosphere and reacted for 3 h. To this reaction mixture (contains compound **2,** mass obtained 4474.8.0 Da, mass calculated 4275.0 Da, purification at this step is not necessary) triethyl phosphine (TEP, 25 μL) was added and kept at room temperature for another 6 h. **3** was precipitated by addition of ice cold 50 % hexanes in methyl *tert*-butyl ether mixture. The precipitate was collected by centrifugation, dried under high vacuum and purified by HPLC using 15-45% acetonitrile gradient in water and 0.05% to give NH_2-e9-c(SH)-oDPRSFL-r9-c(Cy5)-$CONH_2$ (**3,** mass obtained 4386.6 Da, mass calculated 4386.8 Da). To a dry reaction vessel, was added compound **3** (6 mg), DMSO (0.4 mL, anhydrous), m-dPEG12-Mal (2 mg, Quanta Biodesign, product number 10289) and NMM (0.5 μL) under N_2 atmosphere, reacted at room temperature for 12 h and purified by HPLC using 7-50% acetonitrile gradient in water and 0.05% TFA to give **4** mass obtained 5097.6 Da, mass calculated 5097.7 Da). To a dry reaction vessel was added compound **4** (4 mg), Cy7 NHS ester (~0.60 mg, from GE life sciences), DMSO (0.4 mL), and NMM (1μL) and reacted at room temperature for 24 h. The product was purified by HPLC using 15-45% acetonitrile gradient in water and 0.05% TFA to give $RACPP_{DPRSFL}$ (**5**, mass obtained 5762.0 Da, mass calculated = 5762.5 Da, Figure S4a,).

All the other RACPPs (**10**, **15**, **20** and **25**) were synthesized according to general *scheme* 1 and followed a similar synthetic protocol as that of $RACPP_{DPRSFL}$(**5**). Analytical HPLC combined with mass spectrometry was used to confirm the identity of the compounds (Tables S1 and S2).

Purity of the final compounds was assessed by analytical HPLC (Figure S5) using 5-55% acetonitrile in water gradient over 25 mins at 1 ml/min flow rate.

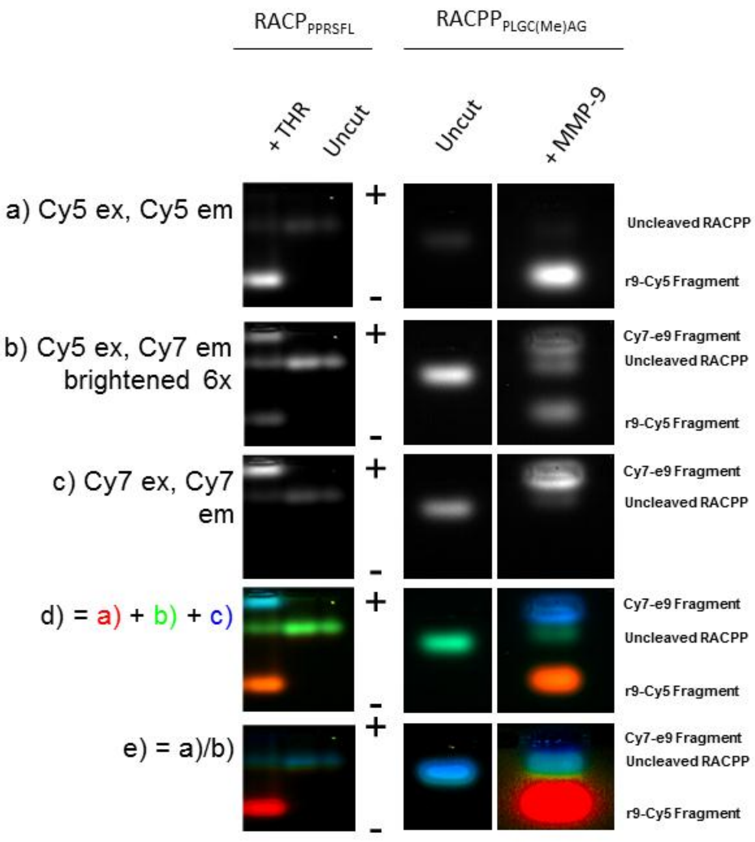

Figure S1. Cleavage of RACPP$_{PPRSFL}$ (**5**) and RACPP$_{PLGC(Me)AG}$ (**20**) by 100 nM MMP-9 or thrombin. Digested peptides were separated on 4% agarose gels in 50 mM pentaethylenehexamine-acetate at pH 5.6. Gels were imaged for a) Cy5 fluorescence, b) Cy7 fluorescence upon exciting Cy5, brightened 6-fold, and c) Cy7 fluorescence. Panel d) is an overlay of a) in red, b) in green, and c) in blue. Panel e) is a pseudocolor Cy5/Cy7 emission ratio image of panels a) and b), generated as in Fig. 2 and 3. The two RACPPs were run on separate gels with the electrophoretic polarity shown between them. The thin white separation in the RACPP$_{PLGC(Me)AG}$ gel marks where irrelevant lanes and specular reflections were excised for clarity.

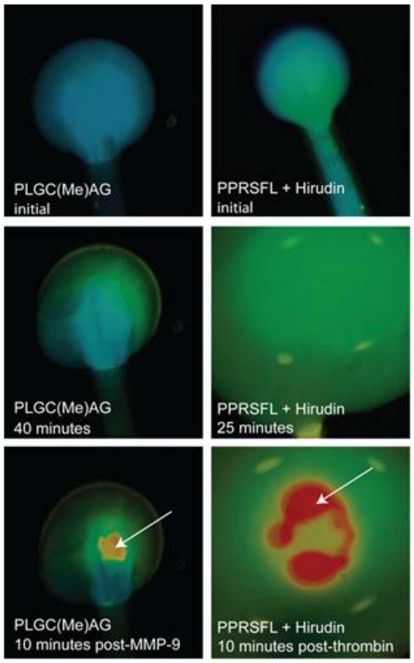

Figure S2. Ratiometric fluorescent images of exudates from tail injuries in mice injected with RACPPs (PLGC(Me)AG, **20** or PPRSFL, **5** with hirudin), before and after the addition of exogenous enzyme. Images were taken 5 minutes after injection (top row), 40 minutes ($RACPP_{PLGC(Me)AG}$) or 25 minutes post injury ($RACPP_{PPRSFL}$) (middle row), or 10 minutes after local addition of either MMP-9 (1 µl of 1.5 µM, bottom left) or thrombin (10 µl of 5µM, bottom right). In each case there was little ratio change until the addition of exogenous enzyme (bottom row). Addition of the appropriate enzyme caused an increase of Cy5/Cy7 emission ratio (arrows). Color scale is identical to Fig. 3.

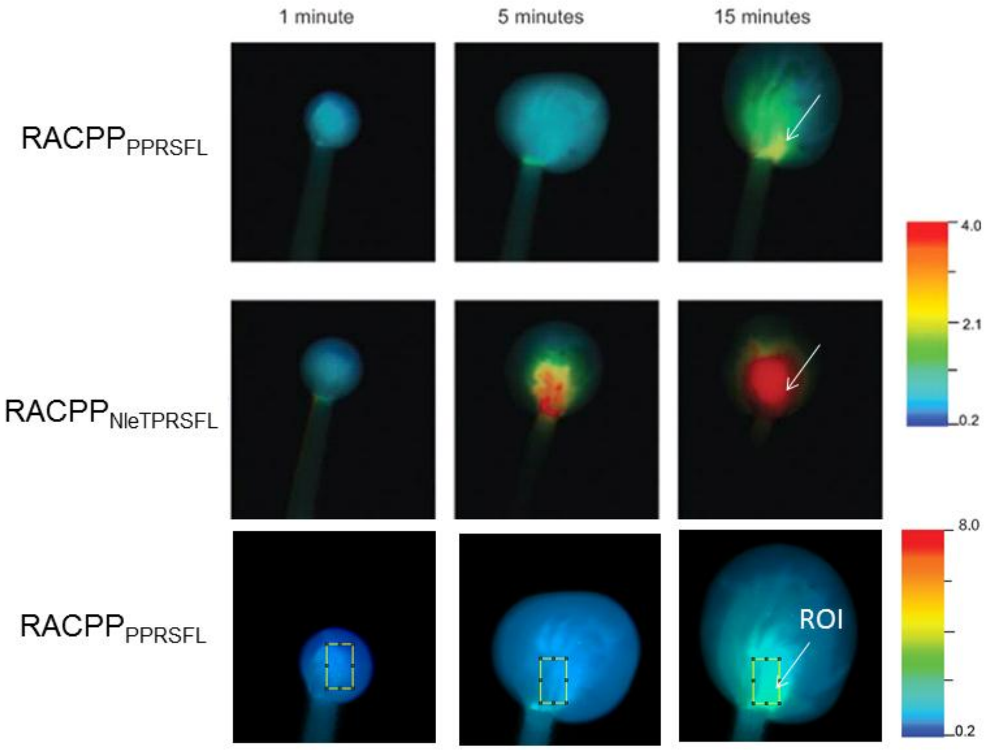

Figure S3. Ratiometric fluorescent images of exudates from tail injuries in mice injected with RACPPs (PPRSFL, 5 or NleTPRSFL, 25). Images were taken 1, 5, or 15 minutes post injury. For quantitation average Cy5 and Cy7 fluorescent intensities were acquired from identical ROIs (highlighted rectangles in figure) and divided to determine Cy5/Cy7 ratios. For the top two rows images were scaled from with Cy5/Cy7 from 0.2 to 4.0, bottom row was scaled from 0.2 to 8.0 as in Figure 4.

Structures of RACPPs:

a)

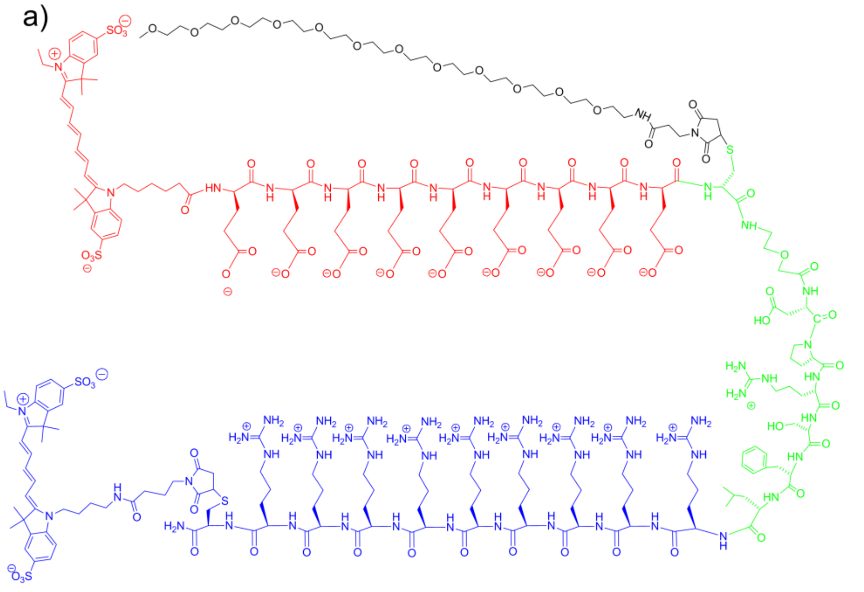

RACPP$_{DPRSFL}$ (**5**)

b)

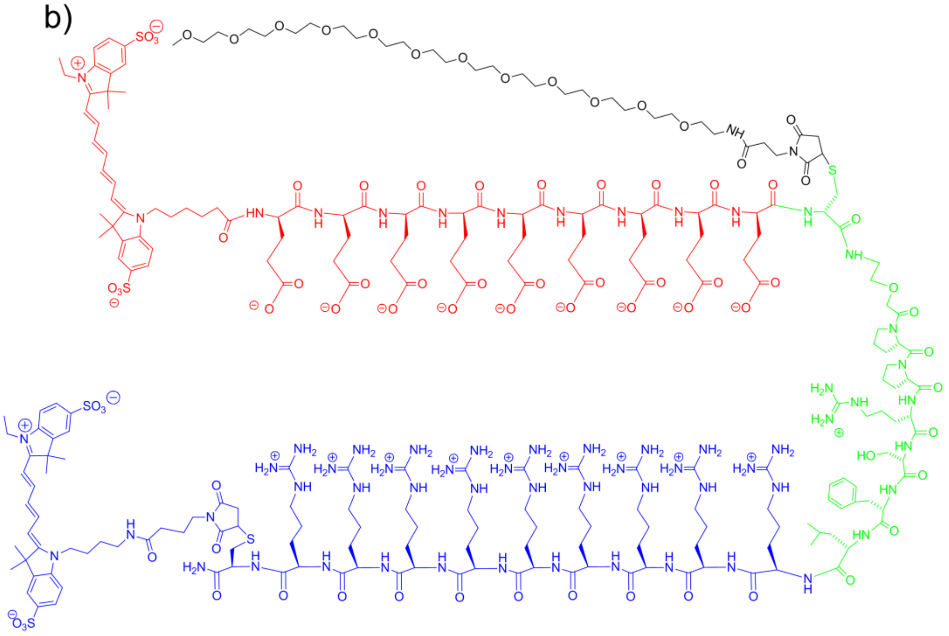

RACPP$_{PPRSFL}$ (**10**)

c)

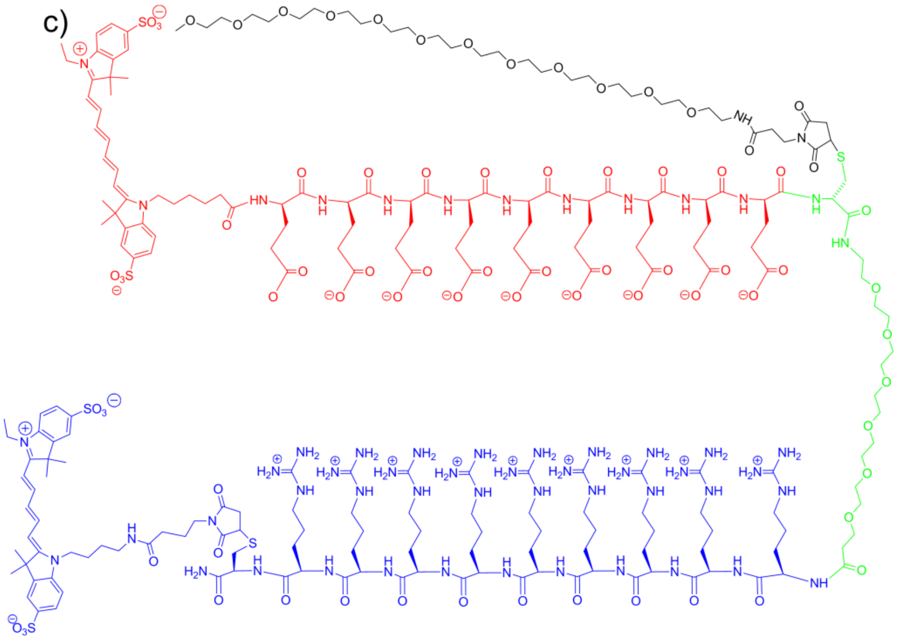

d)

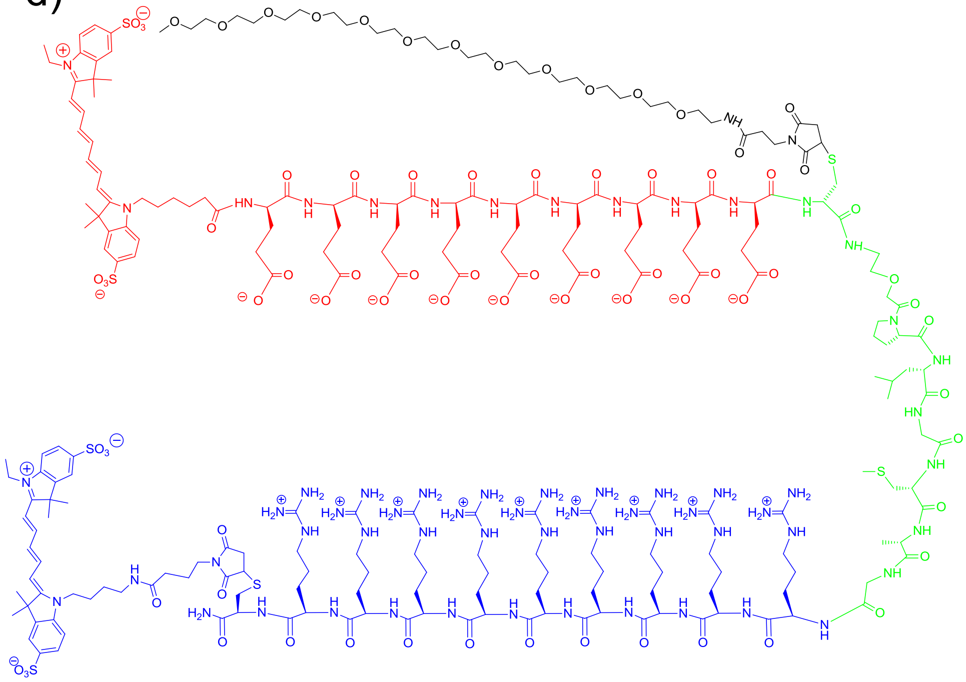

e)

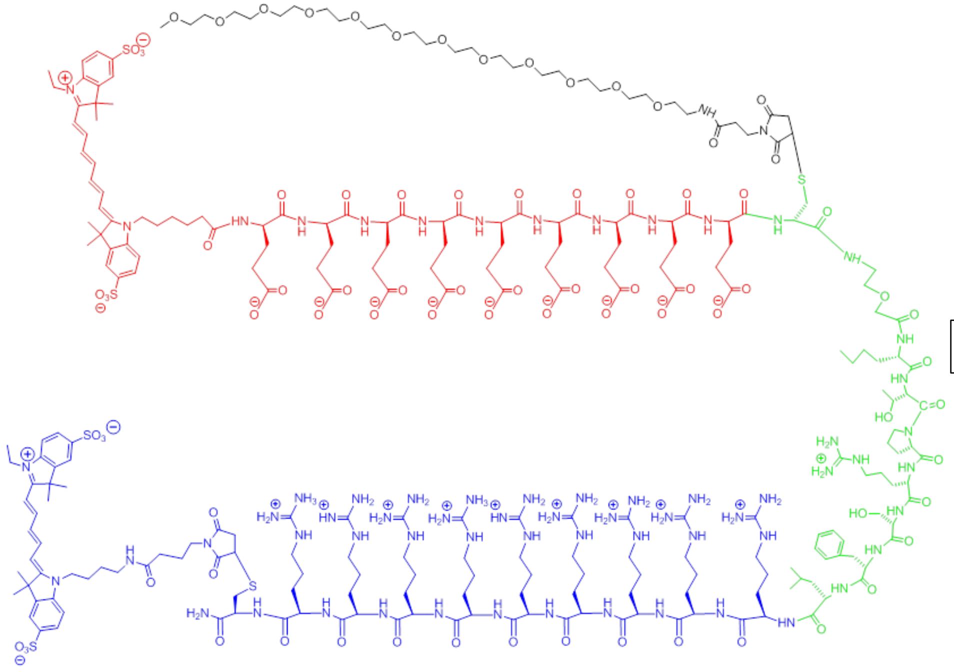

RACPP$_{NleTPRSFL}$ (**25**)

Figure S4. Structures of a) RACPP$_{DPRSFL}$ (**5**), b) RACPP$_{PPRSFL}$ (**10**), c) RACPP$_{peg6}$ (**15**), d) RACPP$_{PLGC(Me)AG}$ (**20**), e) RACPP$_{NLeTPRSFL}$ (**25**).

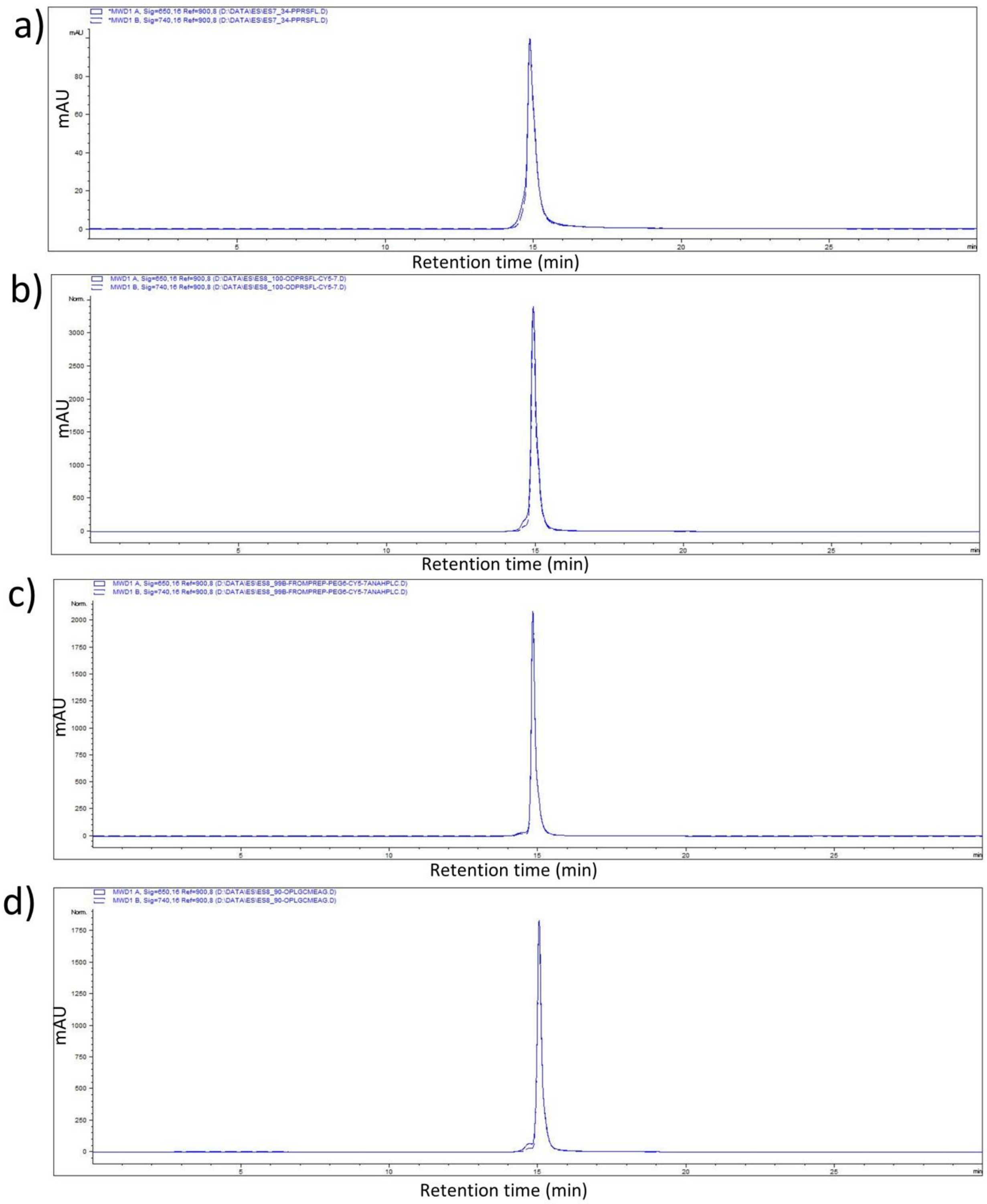

Figure S5: HPLC traces of RACPP$_{DPRSFL}$ (a), RACPP$_{PPRSFL}$(b), RACPP$_{peg6}$(c), RACPP$_{PLGC(Me)AG}$(d) obtained by collecting the absorbance at 650 ± 8 nm (Cy5, solid blue line) and 740 ± 8 nm (Cy7, broken blue line).

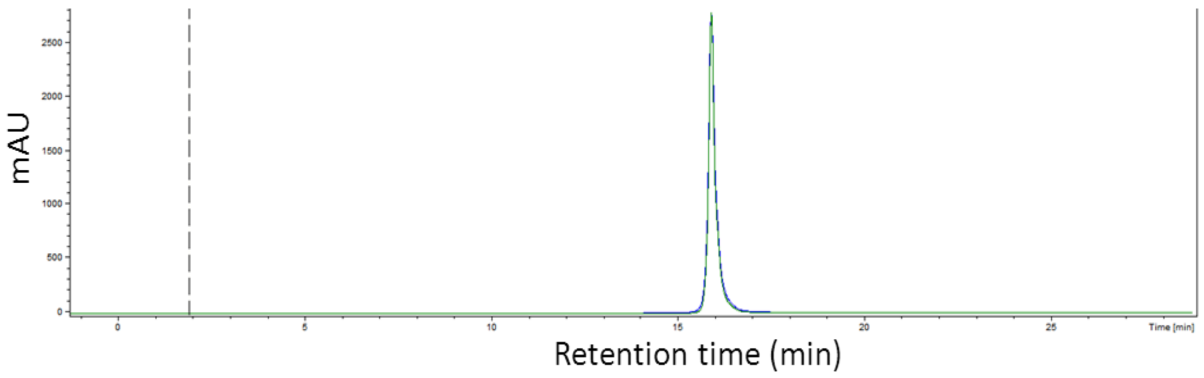

Figure S6: HPLC trace of $RACPP_{NLTPRSFL}$ obtained by collecting the absorbance at 650 ± 8 nm (Cy5, blue line) and 740 ± 8 nm (Cy7, green line).

Table S1. List of compounds and their molecular weights used in this study

Compound	Mass obtained (Da)	Mass Calculated (Da)
NH_2-e9-c(S*t*Bu)oDPRSFL-r9-c-$CONH_2$ (**1**)	3695.7	3696.1
NH_2-e9-c(S*t*Bu)oDPRSFL-r9-c(Cy5)-$CONH_2$ (**2**)	4474.8	4475.0
NH_2-e9-c(SH)oDPRSFL-r9-c(Cy5)-$CONH_2$ (**3**)	4386.6	4386.8
NH_2-e9-c(S-Peg12) oDPRSFL-r9-c(Cy5)-$CONH_2$ (**4**)	5097.6	5097.7
Cy7-NH-e9-c(S-Peg12) oDPRSFL-r9-c(Cy5)-$CONH_2$ (**5**, **$RACPP_{DPRSFL}$**)	5762.0	5762.5
NH_2-e9-c(SS*t*Bu)oPPRSFL-r9-c-$CONH_2$ (**6**)	3678.0	3678.1
NH_2-e9-c(SS*t*Bu)oPPRSFL-r9-c(Cy5)-$CONH_2$ (**7**)	4457.2	4457.0
NH_2-e9-c(SH)oPPRSFL-r9-c(Cy5)-$CONH_2$ (**8**)	4368.4	4368.9
NH_2-e9-c(S-Peg12)oPPRSFL-r9-c(Cy5)-$CONH_2$ (**8**)	5079.4	5079.7
Cy7-NH-e9-c(S-Peg12)oPPRSFL-r9-c(Cy5)-$CONH_2$ (**10**, **$RACPP_{PPRSFL}$**)	5744.4	5744.5
NH_2-e9-c(SS*t*Bu)-peg6-r9-c-$CONH_2$ (**11**)	3214.2	3214.6
NH_2-e9-c(SS*t*Bu)-peg6-r9-c(Cy5)-$CONH_2$ (**12**)	3993.0	3993.5
NH_2-e9-c(SH)-peg6-r9-c(Cy5)-$CONH_2$ (**13**)	3904.8	3905.3
NH_2-e9-c(S-Peg12)-peg6 -r9-c(Cy5)-$CONH_2$ (**14**)	4616.4	4616.1
Cy7-NH-e9-c(S-Peg12)-peg6 -r9-c(Cy5)-$CONH_2$ (**15, $RACPP_{peg6}$)**	5280.8	5281.0
NH_2-e9-c(SS*t*Bu)oPLGC(Me)AG-r9-c-$CONH_2$ (**16**)	3492.6	3492.8
NH_2-e9-c(SS*t*Bu)oPLGC(Me)AG-r9-c(Cy5)-$CONH_2$ (**17**)	4271.6	4271.8
NH_2-e9-c(SH)oPLGC(Me)AG-r9-c(Cy5)-$CONH_2$ (**18**)	4183.3	4183.7
NH_2-e9-c(S-Peg12)oPLGC(Me)AG-r9-c(Cy5)-$CONH_2$ (**19**)	4894.0	4894.5
Cy7-NH-e9-c(S-Peg12)oPLGC(Me)AG-r9-c(Cy5)-$CONH_2$ (**20**, **$RACPP_{PLGC(Me)AG}$**)	5559.6	5559.3
NH_2-e9-c(SS*t*Bu)oNLeTPRSFL-r9-c-$CONH_2$ (**21**)	3794.7	3795.3
NH_2-e9-c(SS*t*Bu)oNLeTPRSFL-r9-c(Cy5)-$CONH_2$ (**22**)	4573.6	4574.2
NH_2-e9-c(SH)oNLeTPRSFL-r9-c(Cy5)-$CONH_2$ (**23**)	4485.5	4486.0
NH_2-e9-c(S-Peg12)oNLeTPRSFL-r9-c(Cy5)-$CONH_2$ (**24**)	5196.0	5196.8
Cy7-NH-e9-c(S-Peg12)oNLeTPRSFL-r9-c(Cy5)-$CONH_2$ (**25**, **$RACPP_{NLeTPRSFL}$**)	5861.4	5861.7

Table S2. Molecular weights of RACPPs obtained using high resolution mass spectrometry

RACPPs	Mono isotopic mass (obtained, Da)	Mono isotopic mass (calculated, Da)
Cy7-NH-e9-c(S-Peg12)oDPRSFL-r9-c(Cy5)-$CONH_2$ (**5**, **$RACPP_{DPRSFL}$**)	5758.6429	5758.6333
Cy7-NH-e9-c(S-Peg12)oPPRSFL-r9-c(Cy5)-$CONH_2$ (**10**, **$RACPP_{PPRSFL}$**)	5740.6650	5740.6591
Cy7-NH-e9-c(S-Peg12)-peg6-r9-c(Cy5)-$CONH_2$ (**15, $RACPP_{peg6}$)**	5277.4158	5277.4147
Cy7-NH-e9-c(S-Peg12)oPLGC(Me)AG-r9-cCy5-$CONH_2$ (**20**, **$RACPP_{PLGC(Me)AG}$**)	5555.5104	5555.5097
Cy7-NH-e9-c(S-Peg12)oNleTPRSFL-r9-cCy5-$CONH_2$ (**25**, **$RACPP_{NLeTPRSFL}$**)	5857.7304	5857.7381

Probe analysis using SDS polyacrylamide gel electrophoresis

Prior to gel electrophoresis 1 µM of each RACPP were cleaved in 150 mM NaCl, 20 mM Tris pH 7.5, 2 mM $CaCl_2$, 1% BSA, 50 nM enzyme at 37°C. Purified thrombin, plasmin, factor Xa, and MMP-9 (activated) were purchased from EMD Chemicals. Samples were mixed with tricine SDS gel loading buffer and heated to 95°C for 5 min before loading in 10-20% tricine gels for electrophoresis. Images were taken on a Maestro multispectral imager (CRI Inc.) with 620 nm excitation and collection for Cy5 (660 to 720 nm) and Cy7 (760 to 830 nm) emission, respectively. Ratiometric images were synthesized by dividing the Cy5 emission by the Cy7 emission. Pseudocolors from blue (ratio minimum) to red (ratio maximum) were assigned using custom designed software.

Probe analysis using PEHA-Acetate agarose gel electrophoresis

$RACP_{PLGC(Me)AG}$ and $RACPP_{PPRSFL}$ were cleaved in 150 mM NaCl, 20 mM Tris pH 7.5, 2 mM $CaCl_2$ or 2 X PBS buffer with 100 nM MMP-9 or thrombin at 37°C respectively. Samples were mixed with PEHA (80 mM pentaethylenehexamine neutralized with acetic acid to make pH 5.6)

buffer containing 15% glycerol, loaded on 4% agarose gel in 50 mM PEHA-Acetate pH 5.6, electrophoretically separated, and imaged as above.

Determination of k_{cat}/K_m

Each probe was diluted in Tris cleavage buffer containing150 mM NaCl, 20 mM Tris pH 7.5, 2mM $CaCl_2$, 1% BSA. Stock solutions of probe were made at to 1.2, 0.6, 0.4, and 0.3 µM. Thrombin, factor Xa were diluted to 10, 40, and 200 nM in Tris cleavage buffer. The plate reader (Safire, Tecan) was set at 37°C in bottom read mode with manual gain setting 110 and kinetic interval of sample measurements every 30 seconds for the first 30 minutes, then every 15 minutes for 2 hours. The excitation and emission wavelengths for Cy5 were set at 630 nm and 680 nm respectively. 50 µl of each concentration of each probe was mixed with 50 µl of each enzyme stock in a black-walled clear-bottom Costar 96 well plate. After mixing, plate reader measurements were started immediately to catch the initial rate of reaction. Percent cleavage was assessed and multiplied by the starting concentration to obtain the total product concentration. The velocity of each reaction was obtained by determination of the slope of the linear portion of the curve on a scatterplot comparing product vs. time. The ratio k_{cat}/K_m was obtained from the reciprocal of the slope of a Lineweaver-Burke plot. Because of concerns about aggregation and intermolecular quenching, substrate concentrations were limited to 1.2 µM, preventing reliable separation of k_{cat}/K_m into numerator and denominator values. For *in vivo* imaging, which is also performed at submicromolar RACPP concentrations, only the ratio of k_{cat} to K_m matters, not their separate values.

Reference:

1) R. B. Majumdar, L. A. Ernst, S. R. Majumdar, C. J. Lewis, A. S. Waggoner, *Bioconjugate Chem*. **1993**, *4*, 105-111.
